# *Abroma augusta* L. (Malvaceae) leaf extract attenuates diabetes induced nephropathy and cardiomyopathy via inhibition of oxidative stress and inflammatory response

**DOI:** 10.1186/s12967-014-0364-1

**Published:** 2015-01-16

**Authors:** Ritu Khanra, Saikat Dewanjee, Tarun K Dua, Ranabir Sahu, Moumita Gangopadhyay, Vincenzo De Feo, Muhammad Zia-Ul-Haq

**Affiliations:** Advanced Pharmacognosy Research Laboratory, Department of Pharmaceutical Technology, Jadavpur University, Kolkata, 700032 India; Biophysics Division, Saha Institute of Nuclear Physics, Kolkata, 700064 India; Department of Pharmacy, University of Salerno, Fisciano, Salerno, 84084 Italy; Office of Research, Innovation and Commercialization, Lahore College for Women University, Lahore, 54600 Pakistan

**Keywords:** *Abroma augusta*, Cardiomyopathy, Nephropathy, Oxidative stress, Streptozotocin-nicotinamide, Type 2 diabetes mellitus

## Abstract

**Background:**

*Abroma augusta* L. (Malvaceae) leaf is traditionally used to treat diabetes in India and Southern Asia. Therefore, current study was performed to evaluate the protective effect of defatted methanol extract of *A. augusta* leaves (AA) against type 2 diabetes mellitus (T2DM) and its associated nephropathy and cardiomyopathy in experimental rats.

**Methods:**

Antidiabetic activity of AA extracts (100 and 200 mg/kg, p.o.) was measured in streptozotocin-nicotinamide induced type 2 diabetic (T2D) rat. Fasting blood glucose level (at specific interval) and serum biochemical markers (after sacrifice) were measured. Redox status, transcription levels of signal proteins (NF-κB and PKCs), mitochondria dependent apoptotic pathway (Bad, Bcl-2, caspase cascade) and histological studies were performed in kidneys and hearts of controls and AA treated diabetic rats.

**Results:**

Phytochemical screening of extracts revealed the presence of taraxerol, flavonoids and phenolic compounds in the AA. T2D rats showed significantly (p < 0.01) elevated fasting blood glucose level. Alteration in serum lipid profile and release of membrane bound enzymes like lactate dehydrogenase and creatine kinase, which ensured the participation of hyperlipidemia and cell membrane disintegration in diabetic pathophysiology. T2DM caused alteration in the serum biochemical markers related to diabetic complications. T2DM altered the redox status, decreased the intracellular NAD and ATP concentrations in renal and myocardial tissues of experimental rats. Investigating the molecular mechanism, activation PKC isoforms was observed in the selected tissues. T2D rats also exhibited an up-regulation of NF-κB and increase in the concentrations of pro-inflammatory cytokines (IL-1β, IL-6 and TNF-α) in the renal and cardiac tissues. The activation of mitochondria dependent apoptotic pathway was observed in renal and myocardial tissues of the T2D rats. However, Oral administration of AA at the doses of 100 and 200 mg/kg body weight per day could reduce hyperglycemia, hyperlipidemia, membrane disintegration, oxidative stress, vascular inflammation and prevented the activation of oxidative stress induced signaling cascades leading to cell death. Histological studies also supported the protective characteristics of AA.

**Conclusions:**

Results suggest that AA could offer prophylactic role against T2DM and its associated reno- and cardio- toxicity.

**Electronic supplementary material:**

The online version of this article (doi:10.1186/s12967-014-0364-1) contains supplementary material, which is available to authorized users.

## Introduction

Diabetes mellitus (DM) is a chronic metabolic disorder characterized by hyperglycemia which may be due to unusual discharge of insulin and/or resistance to insulin. The disease has a significant effect on social, psychological as well as physical quality of life [1]. Approximately 366 million people are suffering from DM around the world and the incidence of this disease is predicted to be more than double by the year of 2030 [2]. Non-insulin dependent or Type 2 DM (T2DM) is most prevalent among various types of DM, which comprises ~ 90–95% of all diagnosed cases of DM [3]. The persistent hyperglycemia is accountable for the damages of various organs and tissues in diabetic patients. However, diabetic complications are not merely due to hyperglycemia. Previous investigations have indicated that oxidative stress is a key factor in etiology of diabetic complications like cardiomyopathy and nephropathy [4,5]. However, the exact source of oxidative free radicals is yet to be recognized. Mitochondrial dysfunction, advanced glycation end processes and others are believed to be the probable sources [6]. Generation of oxidative free radicals results in oxidative disruption of structural proteins and degradation of membrane-bound-phospholipids. Moreover, reactive free radicals cause fragmentation of DNA, which results apoptotic cell death [7]. Oxidative stress also leads to impairment of endogenous antioxidant enzymes due to their non-enzymatic glycosylation and auto-oxidation [8]. These super-active radicals may also disturb the expressions of several transcription proteins like NF-κB, PKCs, caspases etc. [4]. It is well-known that inflammatory cytokines viz. TNF-α, IL-6 and IL-1β play key roles in the cascade of inflammation and systemic insulin resistance and thereby participate directly in diabetic nephropathy and cadiomyopathy [4]. Therefore, it is important to counteract diabetic pathophysiology through multi-target therapeutic agent. Multi-component plant extract (chemically standardized) could offer this multimodal therapeutic value.

*Abroma augusta* L. (Malvaceae), an evergreen shrub, is found throughout the hot and humid parts of India. Leaves and seeds of *A. augusta* are considered to be edible in India and New Guinea. *A. augusta* has an all-embracing history in Ayurvedic system. Leaves are used as a remedy for diabetes, inflammation, rheumatic pain of joints, uterine disorders, and headache [9-11]. The whole plant contains alkaloids, steroids, triterpenes, flavonoids, megastigmanes, and phenylethanoid glycosides [12]. Since the selected plant species was claimed to possess both anti-diabetic and anti-inflammatory activities, the present investigation was performed to evaluate the protective effect of *A. augusta* leaves on T2DM and its associated pathogenesis in renal and cardiac tissues. Streptozotocin-nicotinamide induced T2DM model on experimental rats was chosen for this study. The effect of test drug on fasting blood glucose level, serum lipid profile and other biochemical markers associated with diabetic pathogenesis was investigated. The protective effect of *A. augusta* leaves on oxidative stress and inflammation mediated pathogenesis was investigated and mechanism by which AA exerts its protective effect was evaluated by estimating the transcription levels of signal proteins. Finally, histopathological studies of kidney and heart were performed to confirm the protective effect of *A. augusta* leaves.

## Material and methods

### Preparation of extract

Mature leaves of *A. augusta* were collected from Howrah district, West Bengal, India in May, 2013. The plant material was authenticated (Ref no. CNH/45/2013/Tech.II/1070) by Dr. V. P. Parsad, Taxonomist, Central National Herbarium, Botanical Survey of India, Shibpur, India. A voucher specimen JU/PT/PC/04/2013 was submitted at Advanced Pharmacognosy Research Laboratory, Department of Pharmaceutical Technology, Jadavpur University, India. The dried powdered leaves (2.5 kg) were macerated with MeOH (4 × 10 l) with continuous agitation. The MeOH extract (312 g, yield ≈ 12.5% w/w) was dissolved in Et_2_O (30°C) for removal of fat and waxes. The residue (AA, 210 g) was subjected in this study.

### Phytochemical analysis

Preliminary phytochemical analysis (TLC studies followed by spraying the chromatogram with specific reagents) revealed presence of triterpenoids, steroids, flavonoids and phenolic compounds in AA. Based on the preliminary phytochemical studies, AA was subjected further for detailed phytochemical profiling. AA (100 g) was extracted with CH_2_Cl_2_ (5 × 3 l) to yielding 26 g of CH_2_Cl_2_ soluble fractions which was enriched with steroids and triterpenoids. CH_2_Cl_2_ fractions were subjected to silica gel-column chromatography using mixtures of n-hexane-EtOAc and EtOAc-MeOH of increasing polarity to yield fractions (A-F). Fraction A (0.38 g) was chromatographed with n-hexane-CH_2_Cl_2_ and CH_2_Cl_2_-MeOH, to yield compound 1 (12 mg). Fraction B (2.5 g) was chromatographed with n-hexane-CH_2_Cl_2_ and CH_2_Cl_2_-MeOH to yield compound 2 (72 mg). Fraction C (0.65 g) and fraction D (0.32 g) also yielded compound 2 of 25 and 11 mg, respectively. Fraction E (10.5) was chromatographed with n-hexane-CH_2_Cl_2_ and CH_2_Cl_2_-MeOH to yield compound 3 (24 mg). RP-HPLC analysis of AA was performed to detect presence of phenolics and flavonoids by comparing with reference phytochemical markers. Chromatographic studies were performed by HPLC (Dionex Ultimate 3000, Germany), having a UV detector and a C-18 RP column (250 × 4.6 mm, particle size 51). The samples were dissolved in HPLC-grade methanol and filtered from a 0.45 μm nylon membrane filter (Pall Life Science, USA). The aliquots of the filtrate were eluted with isocratic solvent mixture comprising methanol:acetonitrile:acetic acid:o-phosphoric acid: water (20:10:1:1:20) for flavonoids and methanol:water:acetic acid (25:74:1) for phenolic compounds with flow rate of 1 ml min^−1^ and detected at 340 and 254 nm respectively.

### Animals

Animal models comprised of male Wistar rats (weight 180 ± 20 g; age 2–3 months). The rats were housed in standard polyprophylene cages under standard lab conditions of 12 h light–dark cycle, temperature (20 ± 2°C), relative humidity (50 ± 15%), standard diet and water ad libitum. The animal experiments were pursued at the Department of Pharmaceutical Technology, Jadavpur University, India (CPCSEA Reg. No. 0367/01/C/CPCSEA). The institutional animal ethical committee instructions as well as principles of laboratory animals care [13] were followed throughout experiment.

### Oral Glucose Tolerance Test (OGTT)

Overnight fasted male Wistar rats were divided into 3 groups of 6 rats each. OGTT was performed by treating the animals with glucose (1.5 g/kg, p.o.) [14]. Just after glucose feeding, two groups of rats were fed with AA (100, 200 mg/kg, p.o., respectively). A group of animals were treated with double distilled water (2 ml/kg, p.o.) and kept as control. Blood glucose levels were measured at 0, 30, 60, and 120 min after glucose treatment with the help of single touch glucometer (Ascensia Entrust, Bayer Health Care, USA). Total glycaemic responses were calculated from respective areas under the curve (AUC) during the 120 min of observation period.

### Comparison between streptozotocin and streptozotocin- nicotinamide model

Overnight fasted male Wistar rats were divided into 3 groups of 6 rats each. One group of animals received single intraperitonial injection (i.p.) of streptozotocin (65 mg/kg) in citrate buffer (pH 4.5). The other group of rats were treated with nicotinamide (110 mg/kg, i.p.) followed by (after 15 min) streptozotocin (65 mg/kg, i.p.) in citrate buffer (pH 4.5) [15]. One group of rats without treatment served as control. After 1 week, animals were subjected to fasting blood glucose level and insulin level measurements. Fasting blood serum glucose level was measured by single touch glucometer (Ascensia Entrust, Bayer Health Care, USA). Serum insulin was also measured by kits (Span Diagnostics Ltd., Surat, India) following manufacturer protocol.

### Induction of T2DM and experimental design

T2DM was induced in overnight fasted rats by a single intraperitoneal injection (i. p.) of streptozotocin (65 mg/kg) in citrate buffer (pH 4.5), 15 min later the administration of nicotinamide (110 mg/kg, i. p.) [15]. After 1 week, animals exhibiting fasting glucose levels between 140–200 mg/dl were screened as type 2 diabetic (T2D) rats and were used for the antidiabetic assay. Animals were divided into 5 groups of 6 rats each as follows:

- Gr I: Normal rats were given double distilled water (2 ml/kg, p.o.) daily for 4 weeks;
- Gr II: T2D control rats were given distilled water (2 ml/kg, p.o.) daily for 4 weeks;
- Gr III: T2D rats were given AA (100 mg/kg, p.o.) daily for 4 weeks;
- Gr IV: T2D diabetic rats were given AA (200 mg/kg, p.o.) daily for 4 weeks;
- Gr V: T2D rats were given standard drug glibenclamide (1 mg/kg, p.o.) [5] daily for 4 weeks.

### Serum biochemical analysis

Single touch glucometer (Ascensia Entrust, Bayer Health Care, USA) was utilized to estimate fasting blood glucose level on day 0, 1, 3, 7, 14, 21 and 28. For estimation of serum biochemical parameters, eppendroff tubes rinsed with anticoagulants were used for collection of blood samples after 28 days. From this blood, serum was separated by centrifugation at 3000 g for 10 min [5]. Enzymatic colorimetric kits (Span Diagnostics Ltd., Surat, India) were used for determination of serum triglyceride and cholesterol profiles while serum insulin was measured by kits (Span Diagnostics Ltd., Surat, India) following manufacturers protocols. Glycosylated hemoglobin was estimated according to method of Nayak and Pattabiraman [16]. Lipid profile (total cholesterol, HDL cholesterol, and triglyceride), alanine aminotransferase (ALT) and aspartate aminotransferase (AST), urea and the membrane leakage enzymes (creatine kinase and lactate dehydrogenase) were assessed by standard kits (Span Diagnostic Limited, India) following manufacturer protocol.

### Organs’ biochemical analysis

The rats were anesthetized and sacrificed by cervical dislocation after 28 days. The organs (heart and kidney) were excised, cleaned and washed with ice-cold saline (pH 7.4). The organs were homogenized in Tris–HCl (0.1 M)-EDTA buffer (pH 7.4, 0.001 M) and centrifuged at 12,000 g for 30 min at 4°C. Biochemical parameters were assessed from collected supernatant. The intracellular reactive oxygen species (ROS) production was measured by the method of LeBel and Bondy [17] with a minor modification introduced by Kim *et al.* [18]. Fluorescence spectrometer (Hitachi, F4500) set at excitation wavelength of 488 nm and emission wavelength of 510 nm was used for measurement of 2', 7'dichloro-fluorescein (DCF) formation. The extent of lipid peroxidation was measured by evaluating thiobarbituric cid reactive substances (TBARS) following the protocol of Ohkawa *et al.* [19]. Protein carbonylation was estimated by method of Uchida and Stadtman [20]. Co-enzymes Q (Q_9_ and Q_10_) were isolated and estimated according to the method of Zhang *et al.* [21]. Reduced glutathione (GSH) level was measured by Hissin and Hilf’s method [22]. Activity of antioxidant enzymes like catalase (CAT), superoxide dismutase (SOD), glutathione peroxidase (GPx), glutathione-6-phosphate dehydrogenase (G6PD) and glutathione reductase (GR) was estimated by the methods reported by Ghosh *et al.* [23]. Homogenates were screened for NAD by colometric method [24] while intracellular ATP concentrations were assessed by commercial kits (Abcam, Cambridge, USA) following manufacturer protocol.

### Immunoblotting

SDS-PAGE (10%) was used for separation of proteins from 20 μg protein sample and transferred into nitrocellulose membrane. These membranes were blocked (blocking buffer containing 5% non-fat dry milk) for 1 h at room temperature and later incubated with primary antibodies (anti-rat antibodies produced in rabbit) viz. anti-Bcl-2 (1:500), anti-Bax (1:500), anti-caspase 3 (1:1000), anti-PKC-α (1:500), anti-PKC-β (1:500), anti-PKC-δ (1:500), anti-PKC-ε (1:500), anti-NF-κB (1:1000), and anti-β-actin (1:1000) antibodies at 4°C overnight. Tris-Buffered Saline with 0.1% Tween 20 (pH 7.6) was used for washing of membranes for 15 min, incubated with appropriate HRP conjugated secondary antibody (anti-rabbit antibodies produced in goat) at 1:2000 dilutions at room temperature for 2 h and developed by HRP substrate 3, 3'- diaminobenzidine tetrahydrochloride system (Banglore genei, India).

### Assay of inflammatory cytokines

Concentrations of inflammatory cytokines (IL-1β, IL-6, TNF-α) in cardiac and renal tissue samples were analyzed by ELISA. A labsystem integrated EIA Management System iEMS and Delta Soft 32.22 EMS software was used for measurement of optical density.

### Histological studies

Isolated organs from normal and experimental rats were fixed in buffered formalin (10%) and processed for paraffin sectioning. Sections obtained (approx. 5 μm) were stained by hematoxylin and eosin to study the histology of isolated organs [25].

### Statistical analysis

One-way ANOVA was utilized for statistical analysis of data and expressed as mean ± SE followed by Dunnett’s *t*-test using computerized GraphPad InStat (version 3.05), GraphPad software, USA. The values were considered significant when *p* < 0.05.

## Results and discussion

### Phytochemical analysis

The structures of isolated compounds were characterized by analyzing NMR and mass spectrometric data. Compound 1, 2 and 3 were identified as taraxerone, taraxerol and stigmasterol (Additional file 1: Figure S1). The phenolic and flavonoid compound were identified by RP-HPLC and comparing retention time (R_t_) and UV spectra with standard marker compounds. HPLC analysis revealed presence of flavonoids viz. rutin (22.4 mg g^-1DW^, R_t_: 3.0), myricetin (14.1 mg g^-1DW^, R_t_: 3.9), quercetin (19.8 mg g^-1DW^, R_t_: 5.6) and apigenin (19.2 mg g^-1DW^*,* R_t_: 8.1) (Additional file 1: Figure S2), the phenolic compounds viz. gallic acid (17.2 mg g^-1DW^, R_t_: 4.03) and chlorogenic acid (19.1 mg g^-1DW^, R_t_: 7.24) in AA (Additional file 1: Figure S3).

### Effect on OGTT

**Figure 1:**
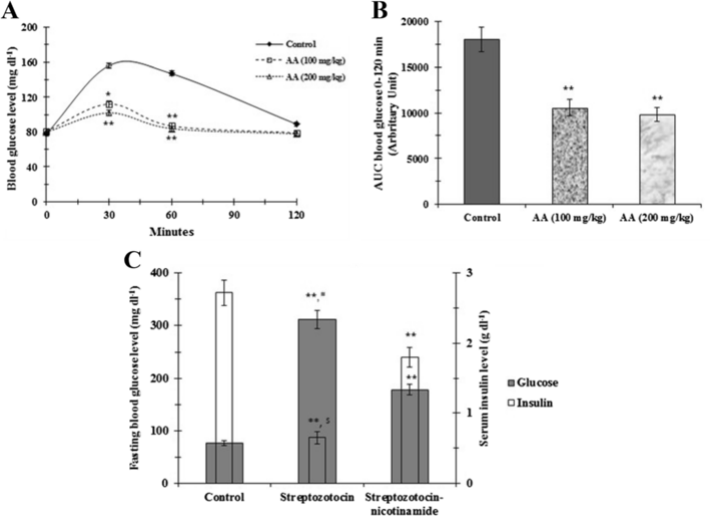
**Effect of AA on oral glucose tolerance test (A); the areas under the curve (AUC) were calculated using the trapezoid method (B); comparison between the effects of streptozotocin (singly) and streptozotocin-nicotinamide (in combination) on adult rats (C).** Data were expressed as mean ± SE (n = 6). *p < 0.05 compared with control group. **p < 0.01 compared with control group. ^#^p < 0.05 compared with streptozotocin-nicotinamide treated rats. ^$^p < 0.01 compared with streptozotocin-nicotinamide treated.

With the intent to assess the effect of AA on systemic glucose homeostasis, OGTT was performed (Figure 1A). The OGTT study revealed that, oral administration of AA (100, 200 mg/kg) significantly (p < 0.05-0.01) reduced blood glucose concentrations between 30–60 min after glucose (1.5 mg/kg) load. The AA also exerted a significant influence on total glycaemic response evidenced by the significant reduction of AUC as compared with control group during an OGTT (Figure 1B).

### Effect of streptozotocin (singly) and streptozotocin in combination with nicotinamide

In this study, the effect of streptozotocin (singly) and streptozotocin in combination with nicotinamide was compared (Figure 1C). Both treatments offered a significant elevation of fasting blood glucose level (p < 0.01) with concomitant depletion (p < 0.01) of serum insulin level, when compared with normal rats. However, a single intraperitoneal injection (i.p.) of streptozotocin (65 mg/kg) caused nearly complete destruction of β-cells of pancreas resulting an elevated fasting blood glucose level (~270-360 mg dl^−1^) with a sharp depletion of insulin level (~0.6 μg dl^−1^). On other hand, the rats exposed to nicotinamide prior to streptozotocin exhibited a blood glucose level ranging between ~160-200 mg dl^−1^ and insulin level of ~ 1.8 μg dl^−1^ after day 7. The statistical comparison between streptozotocin and streptozotocin-nicotinamide treated group revealed a significant difference between fasting blood glucose (p < 0.05) and serum insulin (p < 0.01) levels. The experimental outcome would refer that nicotinamide offers a partial protection against β-cytoxic effect of streptozotocin [15]. Masiello et al. [15] claimed that pretreatment of nicotinamide offered ~ 40% preservation of pancreatic insulin stores and developed T2DM in adult rats. Therefore, streptozotocin-nicotinamide model has been chosen for this study to evaluate oral hypoglycemic effect of AA.

### Effect on fasting blood glucose level and serum biochemical parameters

A significantly (p < 0.01) high fasting blood glucose level (170–190 mg/dl) was observed in T2DM rats. Maintenance of blood glucose level within normal range is a primary approach during T2DM treatment. AA (100 and 200 mg/kg) treatment could significantly reduce fasting blood glucose level in T2D rat with maximum reduction of ~ 32.2% (p < 0.01) and ~ 40.5% (p < 0.01) on day 28, respectively. The standard drug glibenclamide (1 mg/kg) exhibited maximum reduction of ~ 48.3% (p < 0.01) on day 28 (Table 1). Biological effects of AA on serum biochemical parameter were shown in Table 2. T2DM is associated with hyperlipidemia [26]. Investing the lipid profile, significantly (p < 0.01) high levels of total cholesterol, triglycerides with concomitant low HDL cholesterol (p < 0.01) level were observed in the T2D rats, which could suggest the association between hyperglycemia and hyperlipidemia. A significant (p < 0.01) reduction of serum insulin content was observed in T2D rats. It is well-recognized that lipo-protein lipase is activated by insulin which hydrolyzes triglycerides under normal physiological conditions [5]. Therefore, impairment of insulin secretion and/or responsiveness could be the cause of hyperlipidemia in T2DM. However, AA could significantly reinstate the serum lipids (p < 0.05-0.01) and insulin (p < 0.05) levels near to the normalcy. The experimental observation could suggest that AA exerts anti-hyperglycemic and anti-hyperlipidemic effects through promoting insulin secretion. The increase in blood glucose level is responsible for the increased glycosylation of a number of proteins including haemoglobin [4,27]. In this study, T2DM rats exhibited a significantly (p < 0.01) elevated glycosylated-haemoglobin level. AA (200 mg/kg) treatment could significantly (p < 0.05) reverted the glycosylated-haemoglobin level near to the normal status. A reduction of glycosylation of haemoglobin is accountable to the anti-hyperglycemic effect of AA. Type 2 diabetic rats exhibited significantly (p < 0.01) increased levels of serum AST, ALT and urea as compared with normal control group. An increase in ALT and AST levels in blood may indicate degenerative and necrotic changes in livers (not included in this manuscript), kidneys and heart [7,28,29]. An increased serum level of urea may be associated with protein catabolism and kidney dysfunction [30]. Treatment with AA could significantly lower the levels of serum ALT and urea as compared with diabetic control rats, while, no significant effect was observed in AST level. Increased creatinine kinase and lactate dehydrogenase content are the major indicators of cellular damage [31]. Usually, these membranes bound enzymes leak out into the plasma during cellular injury due to disintegration of contractile elements and sarcoplasmic reticulum [31]. In present investigation, both lactate dehydrogenase and creatinine kinase levels in the sera were significantly (p < 0.01) elevated in T2D animals (Table 2). However, treatment with AA (200 mg/kg) could significantly (p < 0.05) revert the levels of lactate dehydrogenase and creatinine kinase as compared with T2D control rats. In this experiment, C-reactive protein activities were significantly up-regulated in diabetic animals. It is reported that the incidence of diabetes is linked with higher levels of various inflammatory parameters like C-reactive proteins [4]. An elevated level of serum C-reactive proteins vindicated the inflammation mediated damage of critical tissues. Treatment with AA could significantly (p < 0.05-0.01) reduce the C-reactive protein levels in a dose-dependent manner.

**Table 1:** **Effect of AA on fasting blood glucose level of T2D rats**

**Groups**	**Fasting blood glucose level (mg dl** ^**−1**^ **) in days**						
	**0**	**1**	**3**	**7**	**14**	**21**	**28**
Gr I	76.48 ± 3.74	76.04 ± 4.08	76.21 ± 3.99	75.84 ± 3.62	75.63 ± 2.63	77.22 ± 2.87	76.43 ± 3.12
Gr II	171.90 ± 6.99^#^	172.28 ± 6.91^#^	177.44 ± 7.45	180.77 ± 6.82^#^	184.13 ± 7.26^#^	187.15 ± 6.15^#^	190.47 ± 6.03^#^
Gr III	170.63 ± 9.02^#^	168.08 ± 8.76^#^	146.33 ± 9.77*	142.64 ± 9.38**	138.88 ± 4.08**	133.27 ± 3.04**	129.10 ± 3.39**
Gr IV	173.22 ± 5.46^#^	165.67 ± 7.39^#^	142.40 ± 5.64**	136.08 ± 6.56**	126.64 ± 4.82**	121.47 ± 5.27**	113.39 ± 4.70**
Gr V	175.32 ± 7.94^#^	168.88 ± 8.85^#^	138.84 ± 5.59**	125.71 ± 5.75**	113.16 ± 4.49**	108.45 ± 3.73**	98.40 ± 2.83**

Data were expressed as mean ± SE (n = 6). ^#^ *p* < 0.01 compared with normal control group. *p < 0.05 compared with diabetic control group.

**p < 0.01 compared with diabetic control group. Gr I: Normal; Gr I: Normal; Gr II: T2D control, Gr III: T2D + AA (100 mg/kg, p.o.); Gr IV: T2D + AA (200 mg/kg, p.o.); Gr V: T2D + glibenclamide (1 mg/kg, p.o.).

**Table 2:** **Effect of AA on serum biochemical parameters of T2D rats**

**Parameters**	**Gr I**	**Gr II**	**Gr III**	**Gr IV**	**Gr V**
Total cholesterol (mg dl^−1^)	87.12 ± 3.60	131.32 ± 3.73^#^	104.22 ± 5.02*	97.53 ± 3.70**	94.43 ± 3.67**
HDL-cholesterol (mg dl^−1^)	33.10 ± 2.94	18.14 ± 1.13^#^	24.61 ± 2.41	28.98 ± 3.60*	31.38 ± 3.17**
Triglycerides (mg dl^−1^)	76.27 ± 3.78	123.08 ± 6.13^#^	104.22 ± 5.67	96.40 ± 7.10*	82.62 ± 5.60**
Insulin (μg dl^−1^)	2.68 ± 0.18	1.64 ± 0.14^#^	2.02 ± 0.11	2.17 ± 0.10*	2.23 ± 0.14*
Glycosylated haemoglobin (mg g^−1^ haemoglobin)	0.27 ± 0.45	0.67 ± 0.62^#^	0.49 ± 0.73	0.42 ± 0.65*	0.39 ± 0.33**
ALT (IU l^−1^)	69.83 ± 4.16	101.43 ± 7.49^#^	84.55 ± 6.35	79.33 ± 5.13*	73.98 ± 6.13*
AST (IU l^−1^)	45.32 ± 4.09	71.69 ± 4.47^#^	58.91 ± 5.38	54.69 ± 6.81	51.20 ± 6.34*
Urea (mg dl^−1^)	30.63 ± 2.41	65.04 ± 4.99^#^	46.56 ± 3.88**	44.76 ± 3.07**	41.86 ± 3.52**
Lactate dehydrogenase (U l^−1^)	207.52 ± 9.36	294.04 ± 15.24^#^	253.75 ± 15.68	242.82 ± 13.10*	238.33 ± 9.98*
Creatinine kinase (IU mg^−1^ protein)	4.73 ± 0.38	8.21 ± 0.50^#^	6.54 ± 0.57	6.12 ± 0.39*	5.76 ± 0.45**
C-reactive protein (mg dl^−1^)	1.02 ± 0.10	3.24 ± 0.24^#^	2.18 ± 0.24*	2.09 ± 0.32**	1.61 ± 0.19**

Data were expressed as mean ± SE (n = 6). ^#^ *p* < 0.01 compared with normal control group. *p < 0.05 compared with diabetic control group.

**p < 0.01 compared with diabetic control group. Gr I: Normal; Gr I: Normal; Gr II: T2D control, Gr III: T2D + AA (100 mg/kg, p.o.); Gr IV: T2D + AA (200 mg/kg, p.o.); Gr V: T2D + glibenclamide (1 mg/kg, p.o.).

### Effect on body and organs’ weight

In the present study, total body weight and the weights of kidney and heart was measured (Table 3). A significant (p < 0.01) decrease of body weight was noticed in diabetic control animals. Extract treatment in either of doses, though statistically insignificant, but definitely improved body weight of T2D rats. In T2DM, loss of insulin responsiveness prevented utilization of glucose for energy to the cells. Under this situation, body starts burning fat and muscle for energy, which would be accountable for loss of body weight in diabetic control rats. In this study, a significant increase (p < 0.01) in kidney weight was observed in diabetic control group as compared with normal control rats. The enhancement of kidney weight would be accountable to glomerular hypercellularity, increase in mesangium and subsequent closer of glomerulus. Treatment with AA (200 mg/kg) could significantly (p < 0.05) reverse this effect near to the normal status. However, no significant changes were observed in weight of heart in any of experimental animal.

**Table 3:** **Effect of AA on body weight and organ weight of T2D rats**

**Groups**	**Total body weight (g)**	**Kidney weight (g)**	**Heart weight (g)**
Gr I	216.67 ± 7.15	0.95 ± 0.05	0.47 ± 0.03
Gr II	171.67 ± 5.58^#^	1.41 ± 0.08^#^	0.52 ± 0.03
Gr III	188.33 ± 6.91	1.17 ± 0.06	0.53 ± 0.04
Gr IV	192.50 ± 5.44	1.12 ± 0.09*	0.52 ± 0.04
Gr V	200.83 ± 8.60*	1.02 ± 0.06**	0.51 ± 0.02

Data were expressed as mean ± SE (n = 6). ^#^ *p* < 0.01 compared with normal control group. *p < 0.05 compared with diabetic control group. **p < 0.01 compared with diabetic control group. Gr I: Normal; Gr I: Normal; Gr II: T2D control, Gr III: T2D + AA (100 mg/kg, p.o.); Gr IV: T2D + AA (200 mg/kg, p.o.); Gr V: T2D + glibenclamide (1 mg/kg, p.o.).

### Effect on renal and cardiac antioxidant markers

Imbalance in cellular redox status due to oxidative stress participate a crucial role in diabetic pathophysiology [5]. The experimental observation confirmed that renal and cardiac dysfunctions resulting from diabetes were accompanied with an alteration in the redox status within the tissues (Tables 4 and 5). The rate of ROS production in diabetic control group was found to be significantly (p < 0.01) higher than that of the normal animals. The inexorable generation of ROS during T2DM could be correlated with the significantly (p < 0.01) elevated levels of lipid peroxidation (TBARS) and protein carbonylation in T2D rats. Lipid peroxidation and protein carbonyl content are the indication of cell membrane damage and oxidative modification of proteins. Treatment with AA (100 and 200 mg kg^−1^), however, could significantly (p < 0.01) inhibit the rate of ROS production in the selected tissues as compared to diabetic rats. Thereby, AA (100 and 200 mg kg^−1^) could simultaneously alleviate (p < 0.05-0.01) that lipid peroxidation and protein carbonylation in renal and myocardial tissues. Ubiquinones (co-enzymes Q) function as important cellular electron carriers distributed within cell organelles chiefly in mitochondria [32]. Co-enzymes Q_9_ and Q_10_ act as antioxidants through scavenging ROS and thereby inhibit lipid peroxidation. A significant reduction of co-enzymes Q (Q9 and Q10) levels in renal (p < 0.01) and cardiac (p < 0.05) tissues was observed in T2D control rats as compared with normal control animals. AA (200 mg kg^−1^) treatment could significantly (p < 0.05) improve Q9 level in kidneys and Q10 levels in kidneys and hearts in T2D animals. Thiol-based antioxidant system (GSH) contributes in cellular defense against free radicals mediated oxidative damage. It protects cells from ROS and consequently converted into its oxidized form, GSSG [29]. According to the present data, the level of GSH decreased significantly (p < 0.01) due to T2DM in rats as compared with normal animals. Oral administration of AA (200 mg/kg) could ameliorate this alteration in renal (p < 0.01) and cardiac (p < 0.05) tissues as compared with diabetic control animals, which would be perhaps due to strong anti-radical activity of AA. Antioxidant enzymes viz. CAT, SOD, GPx, GST, G6PD and GR are first line of cellular defense against oxidative stress [33]. In present study, T2DM caused significant depletion in antioxidant enzymes in cardiac and renal tissues (Table 5). Excessive generation of ROS is accountable for decreasing the activities of antioxidant enzymes in T2DM. While, AA treatment could significantly restore the activities of antioxidant enzymes. The augmented oxidative stress in T2DM was successfully turned-back by AA supplementation indicating its prophylactic potential against oxidative stress and associated dysfunctions in kidneys and heart of T2D rats. The extract may perform its function by electron-transfer, radical recombination, radical addition and/or by quenching free radicals.

**Table 4:** **Effect of AA on ROS production, lipid peroxidation (TBARS), protein carbonyation, ubiquinones (coenzyme Q9 and Q10) and GSH level of T2D rats**

**Parameters**	**Gr I**	**Gr II**	**Gr III**	**Gr IV**	**Gr V**
**Kidney**					
ROS production (nmol DCF min^−1^ mg^−1^ of protein)	36.84 ± 3.14	75.47 ± 4.56^#^	53.51 ± 6.39**	46.89 ± 4.39**	57.85 ± 3.01*
Lipid peroxidation (TBARS level in μg g^−1^ of tissue)	4.56 ± 0.32	7.20 ± 0.45^#^	5.56 ± 0.57*	5.47 ± 0.33*	5.71 ± 0.49*
Protein cabonylation (nmol mg^−1^ of protein)	8.71 ± 0.72	17.71 ± 1.51^#^	13.20 ± 1.32*	11.17 ± 1.06**	11.36 ± 1.27**
Total coenzyme Q9 (nmol g^−1^ of wet tissue)	148.80 ± 4.31	116.50 ± 5.46^#^	127.33 ± 4.45	137.71 ± 5.57*	143.20 ± 4.61**
Total coenzyme Q10 (nmol g^−1^ of wet tissue)	25.38 ± 1.66	16.88 ± 1.78^#^	21.75 ± 1.87	23.60 ± 1.82*	21.32 ± 1.73
GSH (mg g^−1^ tissue)	25.56 ± 1.52	15.12 ± 0.98^#^	20.08 ± 1.43	22.32 ± 1.87**	23.60 ± 1.61**
**Heart**					
ROS production (nmol DCF min^−1^ mg^−1^ of protein)	25.89 ± 1.46	58.14 ± 3.84^#^	40.77 ± 3.72**	37.83 ± 3.01**	45.94 ± 2.82**
Lipid peroxidation (TBARS level in μg g^−1^ of tissue)	3.55 ± 0.24	6.30 ± 0.37^#^	5.09 ± 0.33*	4.61 ± 0.34**	4.94 ± 0.20*
Protein cabonylation (nmol mg^−1^ of protein)	2.48 ± 0.26	5.09 ± 0.42^#^	3.73 ± 0.27*	3.06 ± 0.22**	3.02 ± 0.31**
Total coenzyme Q9 (nmol g^−1^ of wet tissue)	59.12 ± 2.88	43.01 ± 3.15^$^	51.03 ± 3.94	54.44 ± 3.80	55.46 ± 4.30*
Total coenzyme Q10 (nmol g^−1^ of wet tissue)	26.87 ± 2.45	15.84 ± 1.93^$^	22.42 ± 3.18	25.80 ± 2.77*	25.05 ± 2.42
GSH (mg g^−1^ tissue)	22.13 ± 1.54	12.28 ± 1.78^#^	17.40 ± 2.39	19.60 ± 1.24*	19.31 ± 1.92*

Data were expressed as mean ± SE (n = 6). ^$^ *p* < 0.05 compared with normal control group. ^#^ *p* < 0.01 compared with normal control group. *p < 0.05 compared with diabetic control group. **p < 0.01 compared with diabetic control group. Gr I: Normal; Gr I: Normal; Gr II: T2D control, Gr III: T2D + AA (100 mg/kg, p.o.); Gr IV: T2D + AA (200 mg/kg, p.o.); Gr V: T2D + glibenclamide (1 mg/kg, p.o.).

**Table 5:** **Effect of AA on antioxidant enzymes viz. CAT, SOD, GST, GPx, G6PD and GR of T2D rats**

**Parameters**	**Gr I**	**Gr II**	**Gr III**	**Gr IV**	**Gr V**
**Kidney**					
CAT (U mg^−1^ of protein)	226.34 ± 16.33	153.74 ± 9.11^#^	207.28 ± 12.03*	212.65 ± 13.77*	216.33 ± 17.88*
SOD (U mg^−1^ of protein)	140.32 ± 7.05	98.51 ± 6.16^#^	123.82 ± 6.07*	129.15 ± 6.92**	128.56 ± 6.94*
GST (μmol h^−1^ mg^−1^ of protein)	0.71 ± 0.07	0.46 ± 0.04^$^	0.59 ± 0.05	0.68 ± 0.06*	0.67 ± 0.04*
GPx (nmol min^−1^ mg^−1^ of protein)	68.20 ± 5.91	40.43 ± 5.57^$^	54.20 ± 6.12	59.93 ± 8.63	61.82 ± 6.64
GR (nmol min^−1^ mg^−1^ of protein)	69.81 ± 4.90	41.82 ± 3.31^#^	59.48 ± 5.63	61.63 ± 5.74*	63.85 ± 6.30*
G6PD (nmol min^−1^ mg^−1^ of protein)	133.64 ± 7.07	97.44 ± 5.19^#^	117.65 ± 7.69	125.38 ± 8.57*	126.41 ± 6.45*
**Heart**					
CAT (U mg^−1^ of protein)	287.50 ± 11.61	192.26 ± 10.52^#^	230.63 ± 16.50	255.41 ± 15.18*	254.98 ± 20.62*
SOD (U mg^−1^ of protein)	133.66 ± 7.54	95.14 ± 4.66^#^	115.45 ± 7.67	121.96 ± 7.18*	123.60 ± 7.82*
GST (μmol h^−1^ mg^−1^ of protein)	1.63 ± 0.13	1.07 ± 0.12^#^	1.47 ± 0.10	1.53 ± 0.13*	1.55 ± 0.11*
GPx (nmol min^−1^ mg^−1^ of protein)	81.49 ± 8.08	96.74 ± 7.01^#^	133.45 ± 10.13*	138.62 ± 10.69*	139.69 ± 8.35**
GR (nmol min^−1^ mg^−1^ of protein)	26.87 ± 2.45	50.20 ± 4.29^#^	67.20 ± 4.70	73.28 ± 6.67*	75.08 ± 4.69*
G6PD (nmol min^−1^ mg^−1^ of protein)	101.70 ± 7.04	71.23 ± 4.22^#^	88.48 ± 6.29	94.55 ± 4.03*	92.90 ± 4.43*

Data were expressed as mean ± SE (n = 6). ^$^ *p* < 0.05 compared with normal control group. ^#^ *p* < 0.01 compared with normal control group. *p < 0.05 compared with diabetic control group. **p < 0.01 compared with diabetic control group. Gr I: Normal; Gr II: T2D control, Gr III: T2D + AA (100 mg/kg, p.o.); Gr IV: T2D + AA (200 mg/kg, p.o.); Gr V: T2D + glibenclamide (1 mg/kg, p.o.).

### Effect on DNA fragmentation, NAD and ATP levels

**Figure 2:**
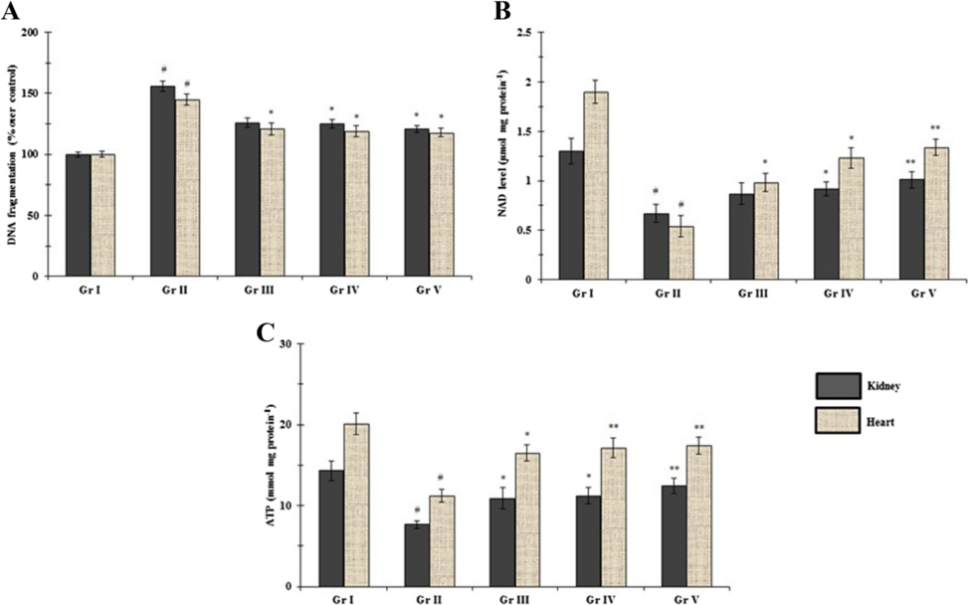
**Effect of AA on DNA fragmentation (A), NAD (B) and ATP (C) levels in the renal and cardiac tissues of type 2 diabetic rats.** Data were expressed as mean ± SE (n = 6). ^#^p < 0.01 compared with normal control group. *p < 0.05 compared with diabetic control group. **p < 0.01 compared with diabetic control group. SE in DNA fragmentation analysis represent the SE of % fragmentation within individual group, while, mean were changed accordingly with respect to 100% in Gr I. Gr I: Normal; Gr II: T2D control, Gr III: T2D + AA (100 mg/kg, p.o.); Gr IV: T2D + AA (200 mg/kg, p.o.); Gr V: T2D + glibenclamide (1 mg/kg, p.o.).

Intercellular redox imbalance plays a key role in DNA damage. The most reactive -OH radical reacts with DNA bases and subtract a hydrogen atom from methyl group of thymine base. As a result, DNA strands are ruptured, or DNA bases are modified abnormally or DNAs are cross-linked. The DNA fragmentation and PARP activation play a pivotal role in cell-death process in diabetic pathophysiology [34]. The DNA fragmentation percentage in renal and myocardial tissues of experimental rats was depicted in Figure 2A. T2DM increased DNA fragmentation in renal and myocardial tissues significantly (p < 0.01) amounting ~ 145 and 156%, respectively. Oral administration of AA (200 mg/kg) inhibited DNA fragmentation in T2D rats, significantly (p < 0.05). In this study, intracellular ATP and NAD levels in the selected tissues were investigated (Figure 2B and C, respectively). It was observed that, hyperglycemia caused significant (p < 0.01) reduction in intracellular NAD and ATP levels in renal and cardiac tissues of diabetic control rats. Activation of inflammatory cytokines induces necrosis via PARP activation during T2DM, which would be accountable for the reduction of intracellular ATP levels. Reduction of ATP level suggested that necrosis simultaneously participate in diabetic pathophysiology. AA treatment, however, could significantly reinstate in cellular ATP (p < 0.05) and NAD (p < 0.05-0.01) levels in both the tissues. Therefore, it would be suggested that AA supplementation could protect renal and myocardial tissues from adverse effects of T2DM by preventing DNA damage and PARP activation.

### Effect on signal proteins

**Figure 3:**
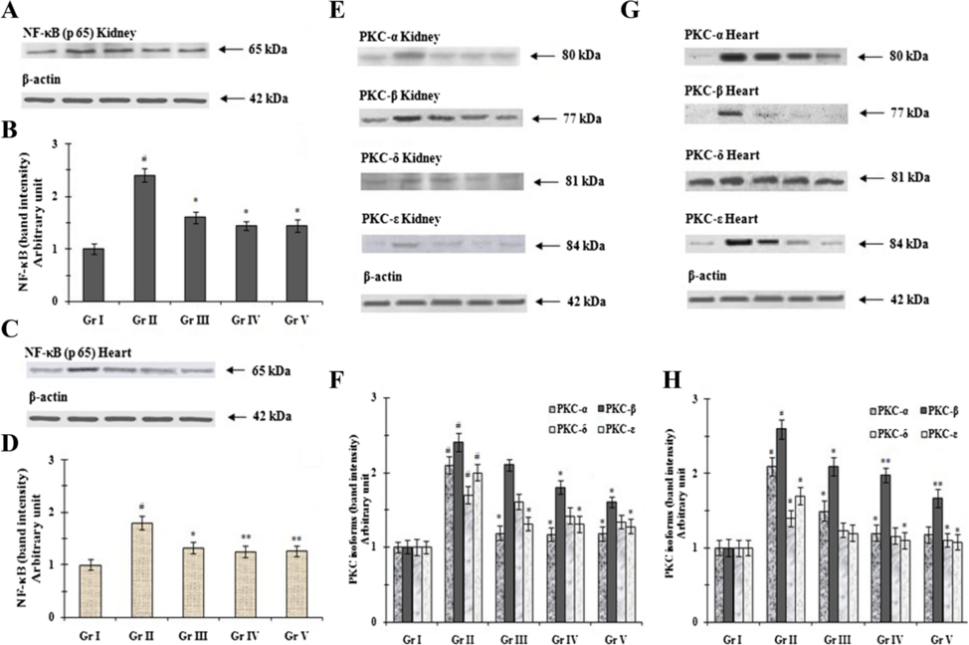
**Western blot analysis of NF-κB (A, B, C, D) and PKC isoforms (E, F, G, H) in the renal (A, B, E, F) and cardiac (C, D, G, H) tissues and of type 2 diabetic rats and AA treated animals.** The relative intensities of bands were determined and the control band was given an arbitrary value of 1. β-actin was used as a loading protein. Data were expressed as mean ± SE (n = 6). ^#^p < 0.01 compared with normal control group. *p < 0.05 compared with diabetic control group. **p < 0.01 compared with diabetic control group. Gr I: Normal; Gr II: T2D control, Gr III: T2D + AA (100 mg/kg, p.o.); Gr IV: T2D + AA (200 mg/kg, p.o.); Gr V: T2D + glibenclamide (1 mg/kg, p.o.).

NF-κB, a ubiquitous transcription factor, gets activated in response to oxidative stress. NF-κB plays integral role in regulation of various inflammatory and immune responses [35]. Therefore the expressions of NF-κB have been investigated in the kidneys (Figure 3A,B) and hearts (Figure 3C,D) tissues of experimental animals under different groups. Interestingly, the expressions of NF-κB were significantly (p < 0.01) increased in renal and myocardial tissues of T2D rats. However, treatment with AA (100 and 200 mg/kg) could significantly (p < 0.05-0.01) reinstate the expressions of NF-κB in the selected tissues near to normalcy. Activation of PKCs within cells during T2DM results in various pathophysiological changes [4]. PKCs are activated via polyol pathway during hyperglycemia [4]. Activated PKC isoforms attribute many abnormal vascular and cellular processes and deregulations viz. endothelial dysfunction, vascular permeability, angiogenesis, apoptosis, changes in vessel dilation, basement membrane thickening, and extracellular matrix expansion, alterations of enzymatic activities and alterations in several transcription proteins [36,37]. Also, PKCs could activate the expression of NF-κB under oxidative stress [38]. Earlier reports revealed that PKC isoforms (PKC-α, β, δ, and ε) are activated in hyperglycemia-mediated nephropathy and cardiomyopathy [37,39]. Different isoforms of PKCs were evaluated through immunoblotting (Figure 3E-H). Immunoblot assays indicated that, T2D rats exhibited significantly (p < 0.01) up-regulated expressions of PKC-α, PKC-β, PKC-δ and PKC-ε in renal (Figure 3E,F) and myocardial (Figure 3G,H) tissues. However, the treatment with AA could significantly (p < 0.05-0.01) reduce the expression of PKC isoforms in diabetic rats.

**Figure 4:**
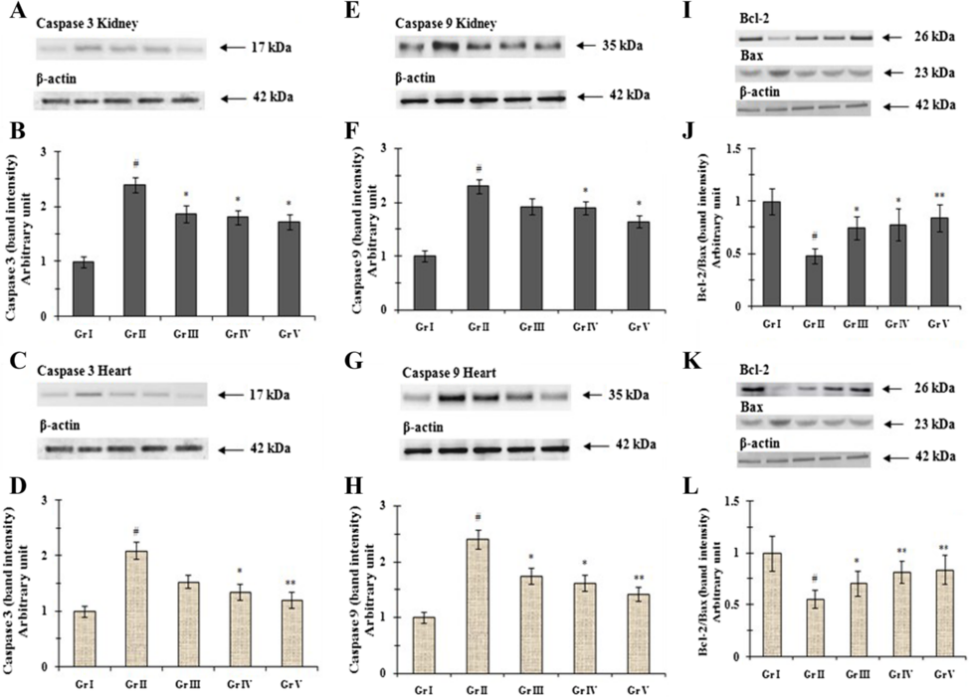
**Western blot analysis of caspase 3 (A, B, C, D), caspase 9 (E, F, G, H), Bcl-2/Bax (I, J, K, L) in the renal (A, B, E, F, I, J) and cardiac (C, D, G, H, K, L) tissues of type 2 diabetic rats and AA treated animals.** The relative intensities of bands were determined and the control band was given an arbitrary value of 1. β-actin was used as a loading protein. Data were expressed as mean ± SE (n = 6). ^#^p < 0.01 compared with normal control group. *p < 0.05 compared with diabetic control group. **p < 0.01 compared with diabetic control group. Gr I: Normal; Gr II: T2D control, Gr III: T2D + AA (100 mg/kg, p.o.); Gr IV: T2D + AA (200 mg/kg, p.o.); Gr V: T2D + glibenclamide (1 mg/kg, p.o.).

Finally, T2D-mediated intrinsic apoptotic cell death pathway in renal and cardiac tissues was studied (Figure 4). Oxidative stress seems to play a crtitical role in mitochondrial dysfunction which is an important early event in the intrinsic pathway of apoptosis. The prime executers of the apoptotic pathways are some pro- and anti-apoptotic proteins and cysteinyl aspartic acid-specific proteases (caspases) [7,40]. Proteins of the Bcl-2 (anti-apoptotic) family act on the mitochondria to regulate the release of cytochrome c and initiate the caspases dependent apoptotic pathway [7]. Bax is a pro-apoptotic protein and can modulate the pro-apoptotic processes by inhibiting the expression of Bcl-2 members. Immunoblot analyses of caspase 3 (Figure 4A-D) and caspase 9 (Figure 4E-H) exhibited significant (p < 0.01) up-regulation of caspases in renal and cardiac tissues of T2D rats, which indicated apoptotic cell damage in selected tissues with the progression of DM. Treatment with AA, however, could significantly (p < 0.05) arrest the over-expression of caspases in renal and myocardial tissues of T2D rats. In this study, T2DM inhibited Bcl-2 significantly and activated the Bax expressions. Therefore, the Bcl-2 to Bax ratio was significantly (p < 0.01) reduced in the kidney (Figure 4I,J) and hearts (Figure 4K,L) of diabetic control rats. Supplementation of AA restored the Bcl-2/Bax ratio thereby preventing the Bax translocation to mitochondrial membranes. The experimental outcome could suggest that the AA treatment could attenuate mitochondria dependent cell death pathway.

### Effect on inflammatory mediators

**Figure 5:**
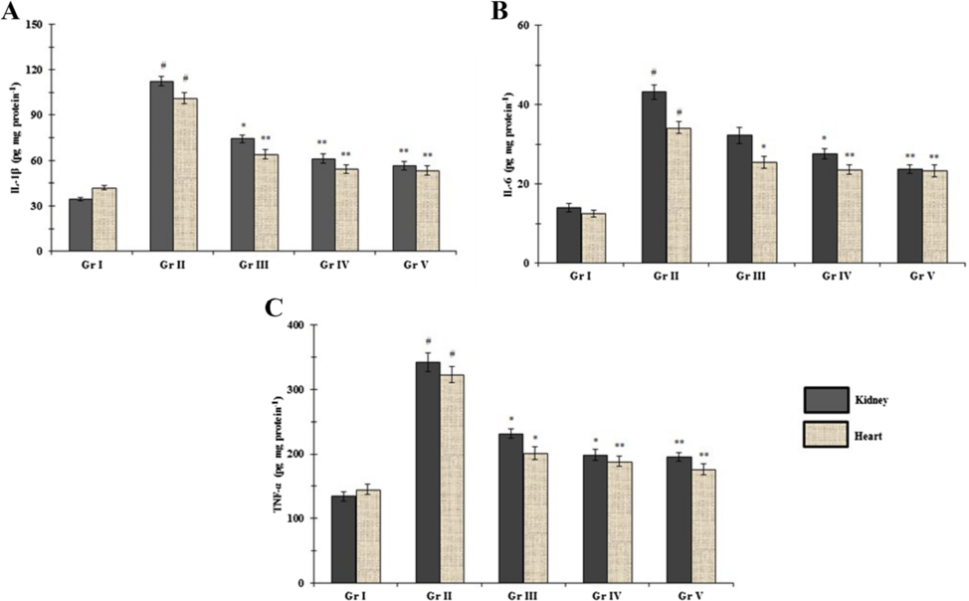
**Effect of AA on pro-inflammatory cytokines IL-1β (A), IL-6 (B) and TNF-α (C) levels in the renal and cardiac tissues of type 2 diabetic rats.** Data were expressed as mean ± SE (n = 6). ^#^p < 0.01 compared with normal control group. *p < 0.05 compared with diabetic control group. **p < 0.01 compared with diabetic control group. Gr I: Normal; Gr II: T2D control, Gr III: T2D + AA (100 mg/kg, p.o.); Gr IV: T2D + AA (200 mg/kg, p.o.); Gr V: T2D + glibenclamide (1 mg/kg, p.o.).

There are mounting evidences that, pro-inflammatory cytokine viz. IL-6, IL-1β and TNF-α play a key role in the development of diabetic cardiomyopathy and nephropathy [4]. An increase in NF-κB expression leads to a consequent increase in the concentrations of aforementioned inflammatory cytokines in diabetic rats which are in-line with previous studies [4,35]. Pro-inflammatory cytokines are chief mediators of inflammatory reaction and stimulate the generation of acute phase proteins. These cytokines function inter-dependently. For example, IL-6 works in connection with IL-1β for synthesis of C-reactive proteins [41]. The TNF-α stimulates hyperlipidemia and hepatic lipogenesis simultaneously reducing the sensitivity to insulin in muscle tissues [42] and finally the necrosis of target organs. In this study, a significant (p < 0.01) increase in the concentrations of pro-inflammatory cytokines was observed in T2DM rats (Figure 5A-C). However, supplementation of AA could significantly reduce the levels of IL-6, IL-1β and TNF-α in the renal and cardiac tissues of diabetic rats in a dose-dependent manner. AA would perhaps act through inhibition of NF-κB expression.

### Effect on histology of organs

**Figure 6:**
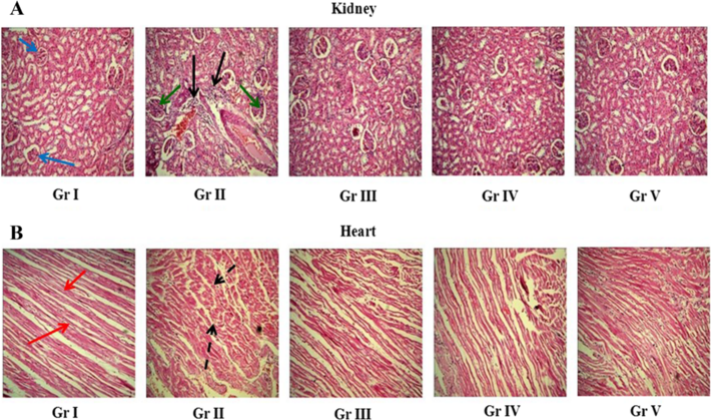
**Histological sections (X 20) of kidneys (Panel A) and heart (Panel B) of normal rats (Gr I), type 2 diabetic rats (Gr II), type 2 diabetic rat treated with AA (100 mg/kg) (Gr III), type 2 diabetic rat treated with AA (200 mg/kg) (Gr IV), type 2 diabetic rat treated with glibenclamide (1 mg/kg) (Gr V).** Blue arrows represent normal glomerular structure. The green arrows showed glomerular hypercellularity. The black arrows indicated tubular necrosis. The red arrows indicated normal radiating pattern of cardiac muscle. The black dotted arrows showed extensive degeneration in cardiac muscle. Gr I: Normal; Gr II: T2D control, Gr III: T2D + AA (100 mg/kg, p.o.); Gr IV: T2D + AA (200 mg/kg, p.o.); Gr V: T2D + glibenclamide (1 mg/kg, p.o.).

Histological study of the kidney tissues indicated that normal cyto-architecture of glomerulus was maintained in control rats (Figure 6, Panel A, Gr I), while, cellular necrosis and glomerular hypercellularity was observed in T2D rats (Figure 6, Panel A, Gr II). Rats receiving AA (100 and 200 mg/kg) restored nearly normal glomerular structures and renal tubules (Figure 6, Panel A, Gr III and IV, respectively). T2DM lead to vast degeneration in cardiac muscles and interstitial fibrosis (Figure 6, Panel B, Gr II) as compared with normal rats (Figure 6, Panel B, Gr I). Supplementation with AA can prevent the disorganization of cell plates and can restore the cardiac cyto-architecture (Figure 6, Panel B, Gr III and IV, respectively) nearly similar to that of normal rats. Glibenclamide, a standard drug (oral hypoglycemic), was used as a reference standard and the effect of AA was compared with glibenclamide (1 mg/kg). The effect of AA at the dose of 200 mg/kg was remained more or less equivalent to that of comparable to that of glibenclamide (1 mg/kg) treated type 2 diabetic animals.

Phytochemical analysis revealed presence of taraxerol which is reported to act as stimulator of glycogen synthesis in 3 T3-L1 adipocytes and as glucose-transport activator [43]. Taraxerol is also reported to reverse insulin resistance [44] and possesses anti-inflammatory properties [45]. Beside taraxerol, AA also contains significant quantity of flavonoids and phenolic constituents which are known phyto-antioxidants [7]. Therefore, a multimodal effect could be achieved due to presence of aforementioned bio-active phytochemicals.

Earlier literatures claimed the prophylactic role of *A. augusta* in diabetes [46]. Islam et al. [10] reported that the aqueous extract of *A. augusta* leaves would be beneficial in managing T2DM. Halim [46] reported the prophylactic role of of aqueous extracts *A. augusta* roots and *A. indica* leaves in combination (1:1). However, both these studies employed alloxan-rats for antidiabetic assay. Despite Islam et al. [10] claimed the effectiveness of *A. augusta* leaves against T2DM but the experimental protocol strongly recommends the development of T1DM in rats which were subjected for their assay. Along with, neither of these published literatures could substantiate any mechanism of *A. augusta* leaves in diabetic pathophysiology nor to establish a correlation between phytochemicals and the observed activity. On other hand, in our study an acceptable model [15] of T2DM has been used to screen antidiabetic effect of *A. augusta* leaves. Supportive data have been presented to show the effect of streptozotocin (singly) and streptozotocin-nicotinamide (combination) on experimental rats to support the experimental model. Significant attempts have made to show the mechanism of action of the test material in diabetic pathophysiology. Finally, the activity has been correlated with phytochemicals present within the *A. augusta* leaves.

**Figure 7:**
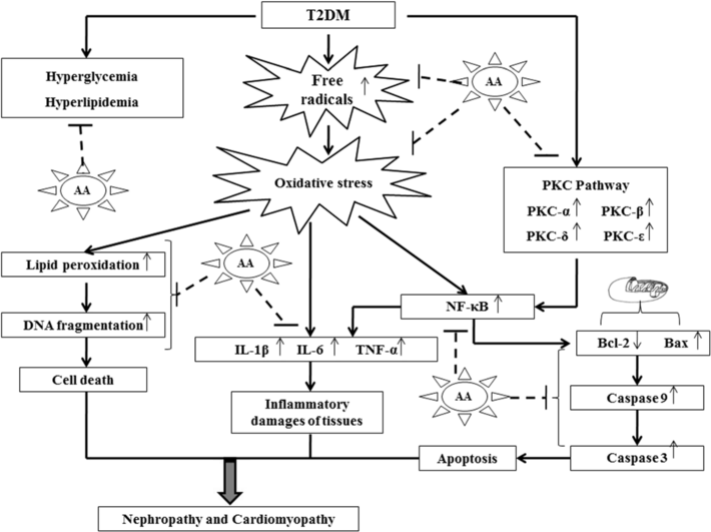
**Schematic diagram of the T2DM mediated renal and cardiac dysfunction and prophylactic role of AA.** Dotted lines represent the therapeutic sites of AA.

In conclusion, this study first demonstrated that AA effectively inhibited hyperglycemia and hyperlipidemia in T2DM, which may be due to presence of sufficient amounts of taraxerol. Supplementation of AA decreased oxidative stress and related inflammatory responses of the heart and kidneys to provide reno-protection and cardio-protection in type 2 diabetic rats. The anti-oxidant effect could be correlated to presence of phenolic compounds and flavonoids, while anti-inflammatory effect could be due to taraxerol. Moreover, AA can reduce the PKCs activation, NF-κB and mitochondria-dependent apoptotic signaling cascades. The overall beneficiary, multimodal effect of AA has been depicted in Figure 7. With these benefits and absence of any adverse effects, AA may be used to treat T2DM and its associated nephropathy and cardio-myopathy. The results provide a justification for future clinical trials of *A. augusta* extract (chemically defined) for the development of oral hypoglycemic agent employing natural resources.

## Additional file

Additional file 1:
**Consists of supportive data on phytochemical investigation of defatted methanol extract of** ***A. augusta*** **extract.** **Figure S1.** described the physical and spectroscopic data of isolated compounds from *A. augusta*. **Figure S2.** depicted the HPLC chromatograms of standard flavonoid markers and flavonoids present within the test extract. **Figure S3.** showed HPLC chromatograms of standard phenolic markers and phenolic compounds present within the test extract.
