# Supplementary material for: Abroma augusta L. (Malvaceae) leaf extract attenuates diabetes induced nephropathy and cardiomyopathy via inhibition of oxidative stress and inflammatory response
Source: J Transl Med. 2015 Jan 16;13:6. doi: 10.1186/s12967-014-0364-1 (PMC4301895; doi:10.1186/s12967-014-0364-1)
Supplement: Additional file 1: — Consists of supportive data on phytochemical investigation of defatted methanol extract of A. augusta extract. Figure S1. described the physical and spectroscopic data of isolated compounds from A. augusta. Figure S2. depicted the HPLC chromatograms of standard flavonoid markers and flavonoids present within the test extract. Figure S3. showed HPLC chromatograms of standard phenolic markers and phenolic compounds present within the test extract. [file 12967_2014_364_MOESM1_ESM.ppt]

## Slide 1
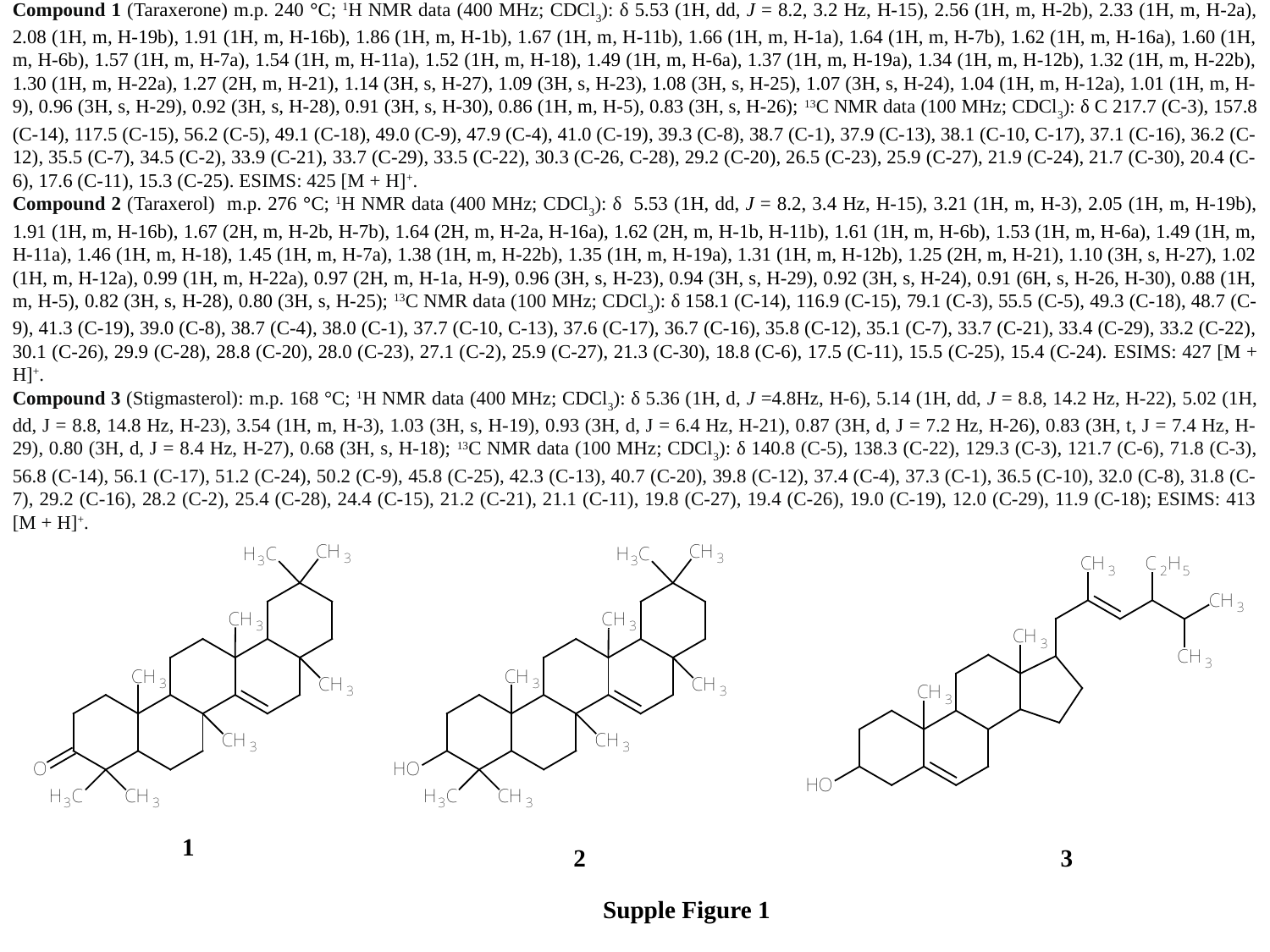

Compound 1 (Taraxerone) m.p. 240 °C; 1H NMR data (400 MHz; CDCl3): δ 5.53 (1H, dd, J = 8.2, 3.2 Hz, H-15), 2.56 (1H, m, H-2b), 2.33 (1H, m, H-2a), 2.08 (1H, m, H-19b), 1.91 (1H, m, H-16b), 1.86 (1H, m, H-1b), 1.67 (1H, m, H-11b), 1.66 (1H, m, H-1a), 1.64 (1H, m, H-7b), 1.62 (1H, m, H-16a), 1.60 (1H, m, H-6b), 1.57 (1H, m, H-7a), 1.54 (1H, m, H-11a), 1.52 (1H, m, H-18), 1.49 (1H, m, H-6a), 1.37 (1H, m, H-19a), 1.34 (1H, m, H-12b), 1.32 (1H, m, H-22b), 1.30 (1H, m, H-22a), 1.27 (2H, m, H-21), 1.14 (3H, s, H-27), 1.09 (3H, s, H-23), 1.08 (3H, s, H-25), 1.07 (3H, s, H-24), 1.04 (1H, m, H-12a), 1.01 (1H, m, H-9), 0.96 (3H, s, H-29), 0.92 (3H, s, H-28), 0.91 (3H, s, H-30), 0.86 (1H, m, H-5), 0.83 (3H, s, H-26); 13C NMR data (100 MHz; CDCl3): δ C 217.7 (C-3), 157.8 (C-14), 117.5 (C-15), 56.2 (C-5), 49.1 (C-18), 49.0 (C-9), 47.9 (C-4), 41.0 (C-19), 39.3 (C-8), 38.7 (C-1), 37.9 (C-13), 38.1 (C-10, C-17), 37.1 (C-16), 36.2 (C-12), 35.5 (C-7), 34.5 (C-2), 33.9 (C-21), 33.7 (C-29), 33.5 (C-22), 30.3 (C-26, C-28), 29.2 (C-20), 26.5 (C-23), 25.9 (C-27), 21.9 (C-24), 21.7 (C-30), 20.4 (C-6), 17.6 (C-11), 15.3 (C-25). ESIMS: 425 [M + H]+.
Compound 2 (Taraxerol) m.p. 276 °C; 1H NMR data (400 MHz; CDCl3): δ 5.53 (1H, dd, J = 8.2, 3.4 Hz, H-15), 3.21 (1H, m, H-3), 2.05 (1H, m, H-19b), 1.91 (1H, m, H-16b), 1.67 (2H, m, H-2b, H-7b), 1.64 (2H, m, H-2a, H-16a), 1.62 (2H, m, H-1b, H-11b), 1.61 (1H, m, H-6b), 1.53 (1H, m, H-6a), 1.49 (1H, m, H-11a), 1.46 (1H, m, H-18), 1.45 (1H, m, H-7a), 1.38 (1H, m, H-22b), 1.35 (1H, m, H-19a), 1.31 (1H, m, H-12b), 1.25 (2H, m, H-21), 1.10 (3H, s, H-27), 1.02 (1H, m, H-12a), 0.99 (1H, m, H-22a), 0.97 (2H, m, H-1a, H-9), 0.96 (3H, s, H-23), 0.94 (3H, s, H-29), 0.92 (3H, s, H-24), 0.91 (6H, s, H-26, H-30), 0.88 (1H, m, H-5), 0.82 (3H, s, H-28), 0.80 (3H, s, H-25); 13C NMR data (100 MHz; CDCl3): δ 158.1 (C-14), 116.9 (C-15), 79.1 (C-3), 55.5 (C-5), 49.3 (C-18), 48.7 (C-9), 41.3 (C-19), 39.0 (C-8), 38.7 (C-4), 38.0 (C-1), 37.7 (C-10, C-13), 37.6 (C-17), 36.7 (C-16), 35.8 (C-12), 35.1 (C-7), 33.7 (C-21), 33.4 (C-29), 33.2 (C-22), 30.1 (C-26), 29.9 (C-28), 28.8 (C-20), 28.0 (C-23), 27.1 (C-2), 25.9 (C-27), 21.3 (C-30), 18.8 (C-6), 17.5 (C-11), 15.5 (C-25), 15.4 (C-24). ESIMS: 427 [M + H]+.
Compound 3 (Stigmasterol): m.p. 168 °C; 1H NMR data (400 MHz; CDCl3): δ 5.36 (1H, d, J =4.8Hz, H-6), 5.14 (1H, dd, J = 8.8, 14.2 Hz, H-22), 5.02 (1H, dd, J = 8.8, 14.8 Hz, H-23), 3.54 (1H, m, H-3), 1.03 (3H, s, H-19), 0.93 (3H, d, J = 6.4 Hz, H-21), 0.87 (3H, d, J = 7.2 Hz, H-26), 0.83 (3H, t, J = 7.4 Hz, H-29), 0.80 (3H, d, J = 8.4 Hz, H-27), 0.68 (3H, s, H-18); 13C NMR data (100 MHz; CDCl3): δ 140.8 (C-5), 138.3 (C-22), 129.3 (C-3), 121.7 (C-6), 71.8 (C-3), 56.8 (C-14), 56.1 (C-17), 51.2 (C-24), 50.2 (C-9), 45.8 (C-25), 42.3 (C-13), 40.7 (C-20), 39.8 (C-12), 37.4 (C-4), 37.3 (C-1), 36.5 (C-10), 32.0 (C-8), 31.8 (C-7), 29.2 (C-16), 28.2 (C-2), 25.4 (C-28), 24.4 (C-15), 21.2 (C-21), 21.1 (C-11), 19.8 (C-27), 19.4 (C-26), 19.0 (C-19), 12.0 (C-29), 11.9 (C-18); ESIMS: 413 [M + H]+.
1
2
3
Supple Figure 1

## Slide 2
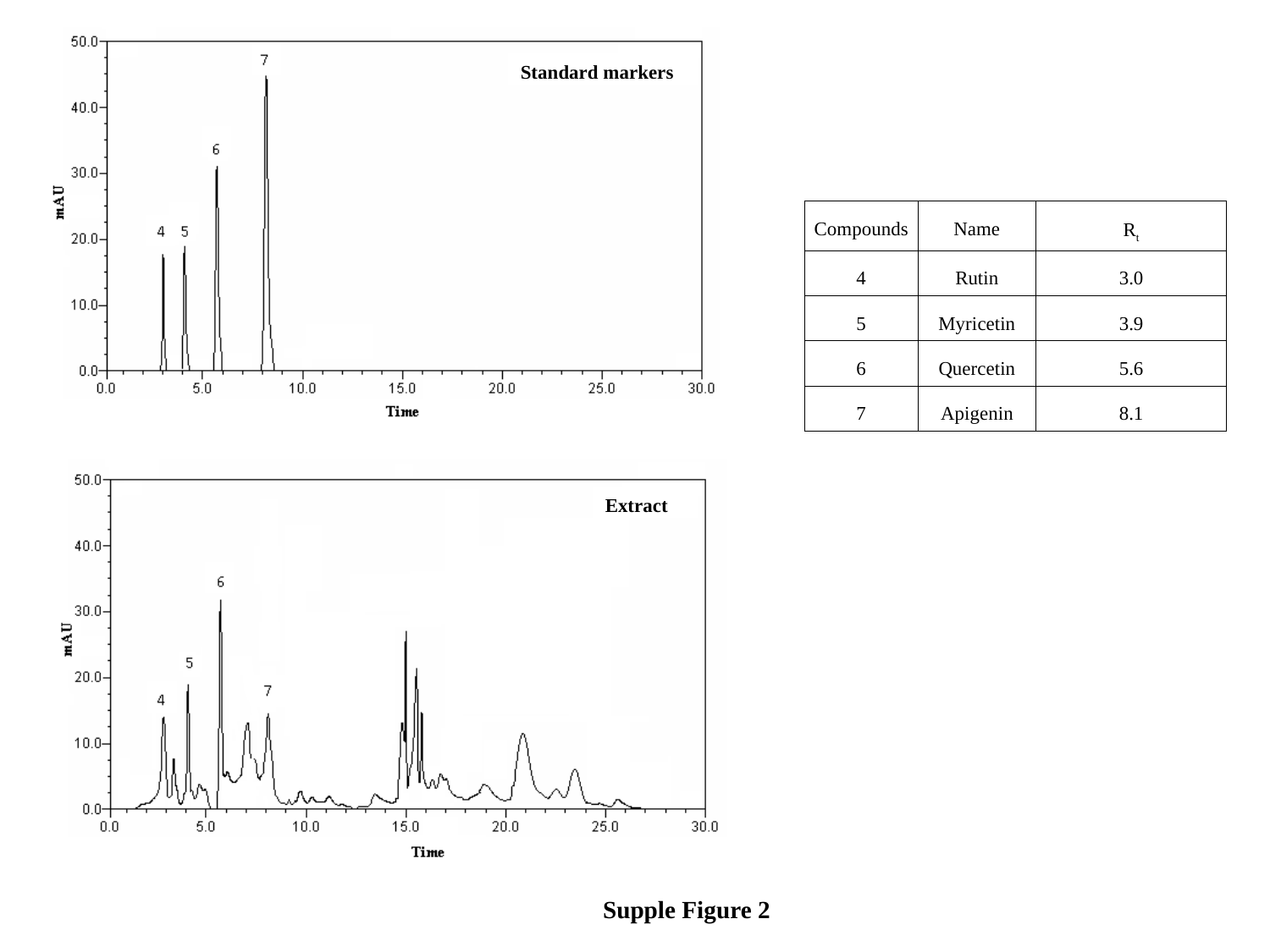

Standard markers
| Compounds | Name | Rt |
| --- | --- | --- |
| 4 | Rutin | 3.0 |
| 5 | Myricetin | 3.9 |
| 6 | Quercetin | 5.6 |
| 7 | Apigenin | 8.1 |
Extract
Supple Figure 2

## Slide 3
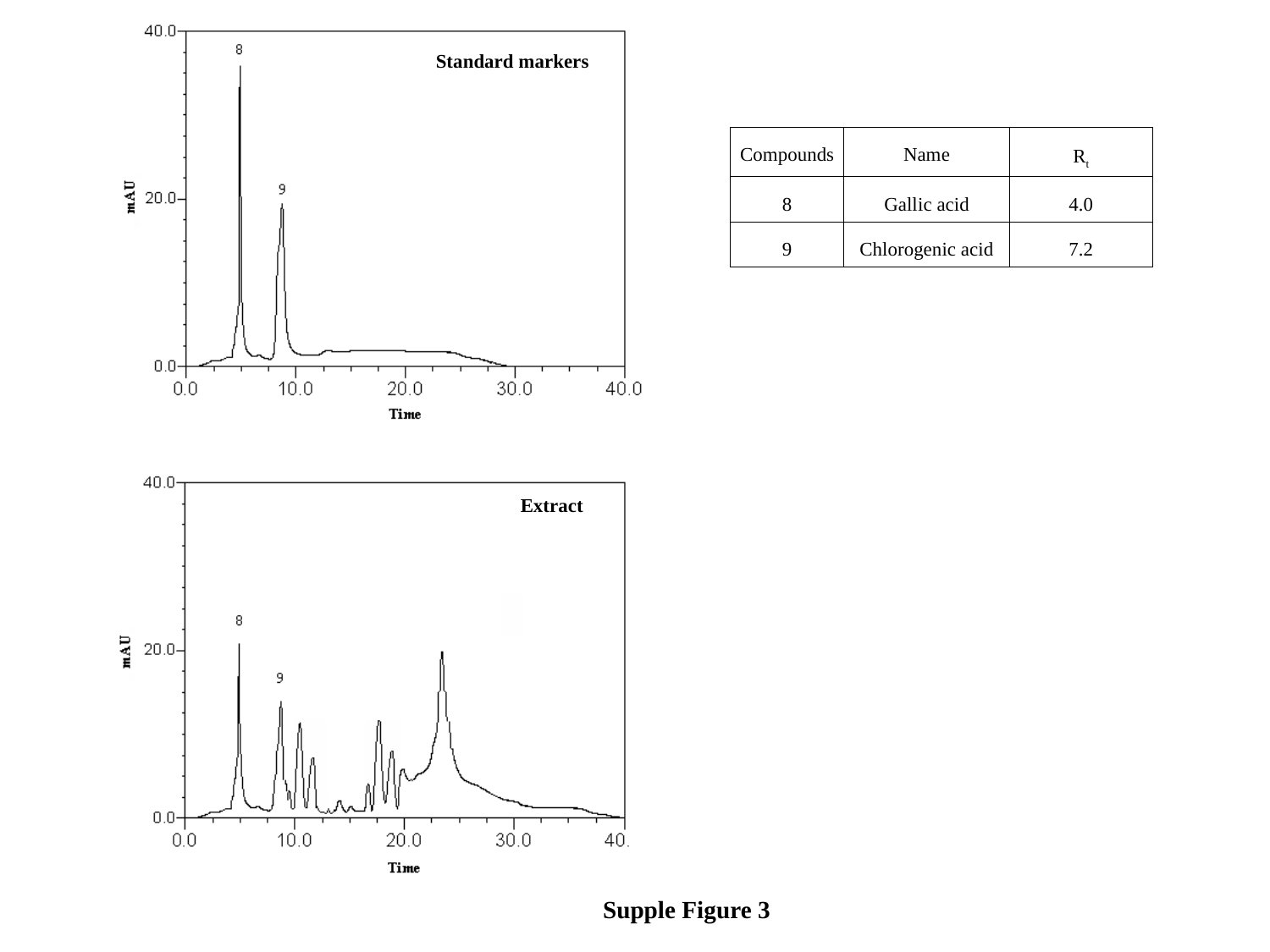

Standard markers
| Compounds | Name | Rt |
| --- | --- | --- |
| 8 | Gallic acid | 4.0 |
| 9 | Chlorogenic acid | 7.2 |
Extract
Supple Figure 3
